# Integrated faecal microbiota and blood metabolic changes following different dietary zinc oxide levels in weaned piglets

**DOI:** 10.1038/s41598-025-03103-7

**Published:** 2025-05-26

**Authors:** Elham Assadi Soumeh, Tina Skau Nielsen, Mette Skou Hedemann, Mihai Victor Curtasu

**Affiliations:** 1 School of Agriculture and Food Sustainability, Faculty of Science, University of Queensland, Gatton Campus, QLD 4343 Australia; 2 Department of Animal and Veterinary Sciences, Aarhus University AU-Viborg, Blichers Allé 20, Tjele, DK-8830 Denmark

**Keywords:** Gut health, Microbiota, Metabolome, Piglets, Zinc, Zoology, Microbiome

## Abstract

This study investigated faecal microbial composition and blood metabolome profile of piglets fed different levels of supplementary zinc oxide (ZnO) after weaning. A dose-response study was conducted with four experimental diets containing 153 (D153), 1022 (D1022), 1601 (D1601), and 2407 (D2407) ppm zinc (Zn) in the feed. At the end of the trial, blood and faeces samples were obtained for analyses. Multivariate analysis of the blood metabolomics dataset and Principal Coordinate Analysis (PCoA) of faecal microbiota data showed that pigs receiving D2407 had a different metabolic and microbial profile to the other groups, whereas no differences were observed in pigs fed with D153, D1022, and D1601. The highest dietary Zn inclusion was associated with significant increase in the abundance of *Clostridium sensu stricto*, *Terrisporobacter*, *Dorea*, and *Prevotellaceae_NK3B31_group* and a decrease in relative abundances of *Methanobrevibacter*, *Treponema*, *Megasphaera*, and *UCG 002 genera*. Pearson’s correlation analysis showed positive correlations between the abundance of *Christensenellaceae R7-group* with amino acids metabolism and production of microbial metabolites. The results suggest that only 2407 ppm Zn altered gut microbiota and modulated blood metabolic profile, which may impact the health status of piglets through specific microbial metabolites.

**Supplementary Information:**

The online version contains supplementary material available at 10.1038/s41598-025-03103-7.

## Introduction

Zinc (Zn), as a co-factor for more than 300 metalloenzymes in the body, is involved in many physiological processes including immune function, growth, and reproduction^1^. Both short and-long term Zn inadequacy or deficiency can result in various symptoms in pigs, including growth retardation, loss of appetite, skeletal abnormalities, parakeratosis, impaired immune function, and impaired gut integrity^2,3^. As an essential micronutrient, piglets require 80 to 100 mg Zn/kg feed^4^ corresponding to a daily Zn intake of between 26.6 and 46.8 mg/day for 5 to 11 kg of bodyweight^5^. However, a recent work showed much higher dietary Zn requirements (1100–1400 ppm) corresponding to a daily Zn requirement of approximately 400 mg Zn for optimum growth in piglets immediately after weaning^6^. In addition to the biological roles of Zn in bodily functions, zinc oxide (ZnO) supplementation at therapeutic doses (2500 ppm) has been a practical nutritional strategy to reduce the incidence and severity of post-weaning diarrhea (PWD) in piglets. Post-weaning diarrhea, one of the most significant challenges in the pig industry, is a condition of gut microbiota dysbiosis and enteric infections that happens due to multiple factors. These include abrupt changes in the diet and environment of piglets immediately after weaning, inadequate digestive enzyme production, stress, poor hygiene, and pathogenic infections^7^. Supplementing dietary ZnO at therapeutic doses after weaning helps mitigating the impact of stress and changes in diet during the weaning process, control *E. coli* infection, and manage PWD^8–10^. However, since it may lead to development of bacterial antimicrobial resistance, environmental issues due to large Zn excretion and sustainability concerns^11^, the European Union banned the use of so-called medicinal dietary ZnO (2500ppm Zn) from June 2022.

Evidence indicates that gut microbiota composition is modulated by high levels of dietary Zn^12,13^. Starke et al. (2014) investigated the effects of low (57 ppm) and high (2,425 ppm) dietary ZnO supplementation in weaned piglets over a 5-week post-weaning period. Diets supplemented with 2,425 ppm Zn, as opposed to 57 ppm, were associated with a significant decrease in *Enterobacteriaceae*, *Escherichia*, and *Lactobacillus spp*. populations. The effects on *Enterobacteriaceae* diminished over time; however, high dietary ZnO exerted a lasting impact on *Lactobacillus* species. The study concluded that elevated dietary Zn inclusion post-weaning induces long-term alterations in gut microbiota composition and metabolic activity^13^. To the best of our knowledge, no study has specifically investigated how varying dietary Zn levels affect blood metabolic profile through the changes in gut microbiota composition, function, and the intermediate microbial metabolites. A recent study has explored the effects of different dietary copper (Cu) levels, combining microbiome and metabolome analyses of intestinal content. The results of that study demonstrated an association between microbial genera and colonic metabolites, showing that different Cu levels altered gut bacteria, impacting carbohydrate and protein metabolism. Similar to Zn, high doses of Cu are used to manage PWD in pigs. These findings underscore the significant influence of nutrition on gut microbiota and microbial metabolites that are involved in host’s metabolism^14^.

It remains unclear at what dietary Zn level it transitions from functioning as a micronutrient to acting as a pharmaceutical agent. The aim of the current study was to investigate gut microbiota and their metabolic activity and associated blood metabolic profile of weaned piglets in response to increasing dietary Zn levels. This research seeks to provide a deeper understanding of the mechanisms through which Zn acts as both a micronutrient and a therapeutic agent.

## Results

### Effect of dietary Zn level on the composition of faecal microbiota

**Fig. 1:**
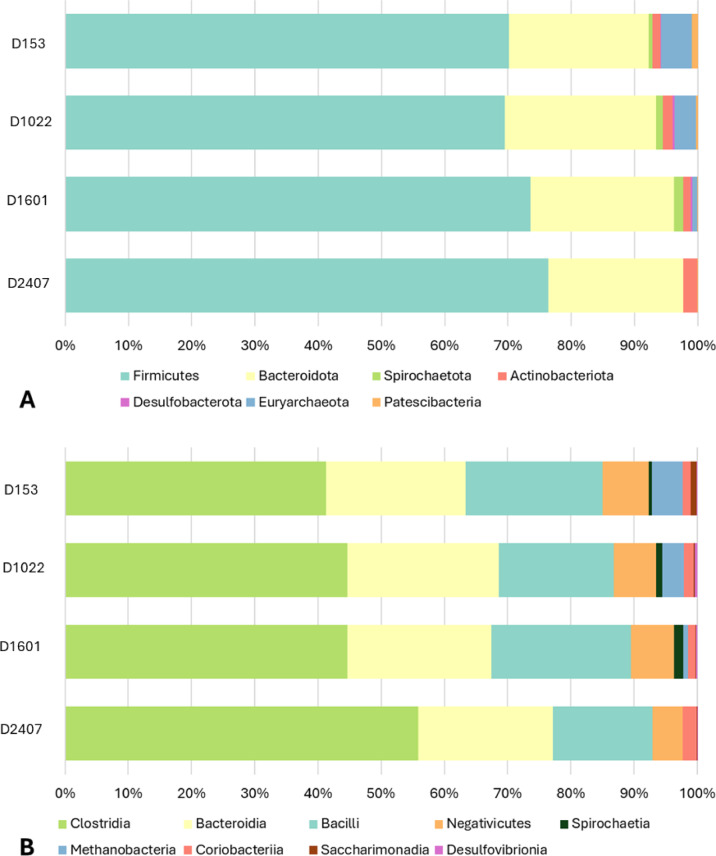
Relative abundance of amplicon sequence variants (ASVs) at phylum (**A**) and Class (**B**) levels between experimental treatments: ZnO 153 ppm (D153), ZnO 1022 ppm (D1022), ZnO 1601 ppm (D1601) and ZnO 2407 ppm (D2407).

Following sequencing, quality control, amplicon clustering, and taxonomic classification, a total of 1,954,605 sequence reads were obtained for all analysed samples and on average 48,865 sequence reads per sample. Supplementary Figure S1 shows the rarefaction plot (the total number of raw sequence reads, and richness obtained per sample and dietary group) and demonstrates that an appropriate sequence depth per sample was achieved, and thus a robustly microbial community was represented in each sample. Profiling of the bacterial abundance revealed that *Firmicutes* and *Bacteroidota* were the dominant taxa at the phylum level and consistent at all four dietary Zn levels, followed by *Actinobacteriota*. Less predominant taxa, such as *Eurychaeota*, *Spirochaeota*, *Desulfobacterota*, *Patescibacteria*, reduced gradually with increasing dietary Zn and disappeared at 2407 ppm Zn (Fig. 1A). At class level, *Spirochaetia*, *Methanobacteria*, *Coriobacteriia*, *Desulfovibrionia* and *Saccharimonadia* seemed to disappear at 2407 ppm Zn (Fig. 1B).

**Fig. 2:**
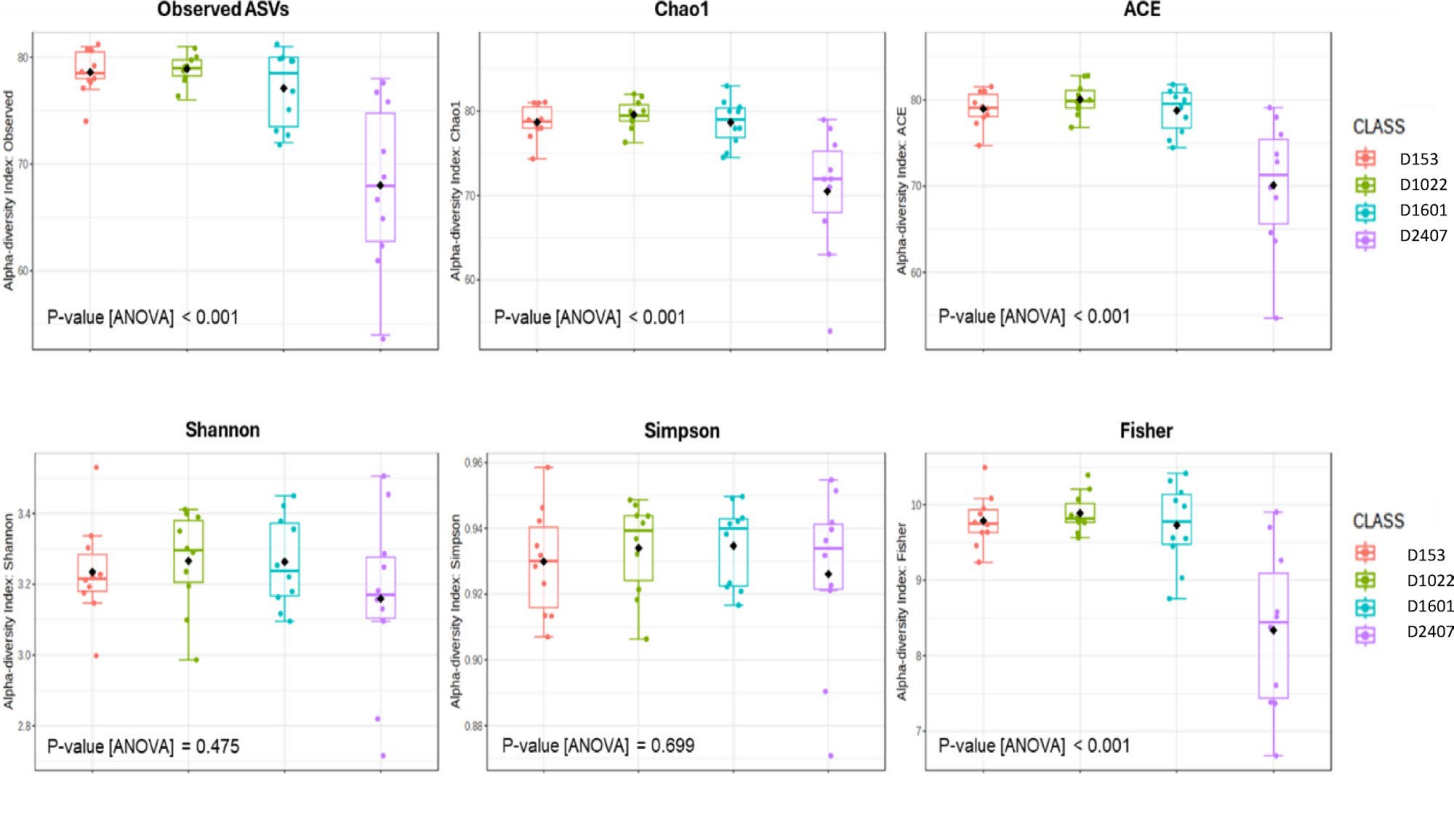
Alpha diversity indexes of faecal microbial profile: observed ASVs, Chao1 index, ACE, Shannon, Simpson, Fisher’ alpha and results of ANOVA comparison between the different dietary ZnO levels.

Parameters characterizing microbial alpha diversity, including, Shannon, Simpson, and Fisher indexes are presented in Fig. 2. Richness parameters, such as, observed amplicon sequence variants (ASVs; amount of unique ASVs found in each sample), ACE and Chao1 showed significant differences between 2407 ppm Zn with a reduced microbial richness compared to diets containing 153, 1022 and 1601 ppm Zn (*p* < 0.001). However, when considering both richness and evenness, the Shannon index and Simpson’s index showed no statistically significant difference between the four dietary Zn levels, but Fisher’s alpha index indicated a significant difference between 2407 ppm ZnO and the lower ZnO levels (*p* < 0.001) as seen in Fig. 2. Evenness measured the relative abundance of different ASVs comprising the richness. Therefore, the results suggest that the microbial profiles shared similar abundance and evenness of species across all levels of dietary ZnO administered. On the other hand, Shannon indexes for the majority of samples were above 3.0, indicating the existence of a highly diverse microbiota per sample^15^.

**Fig. 3:**
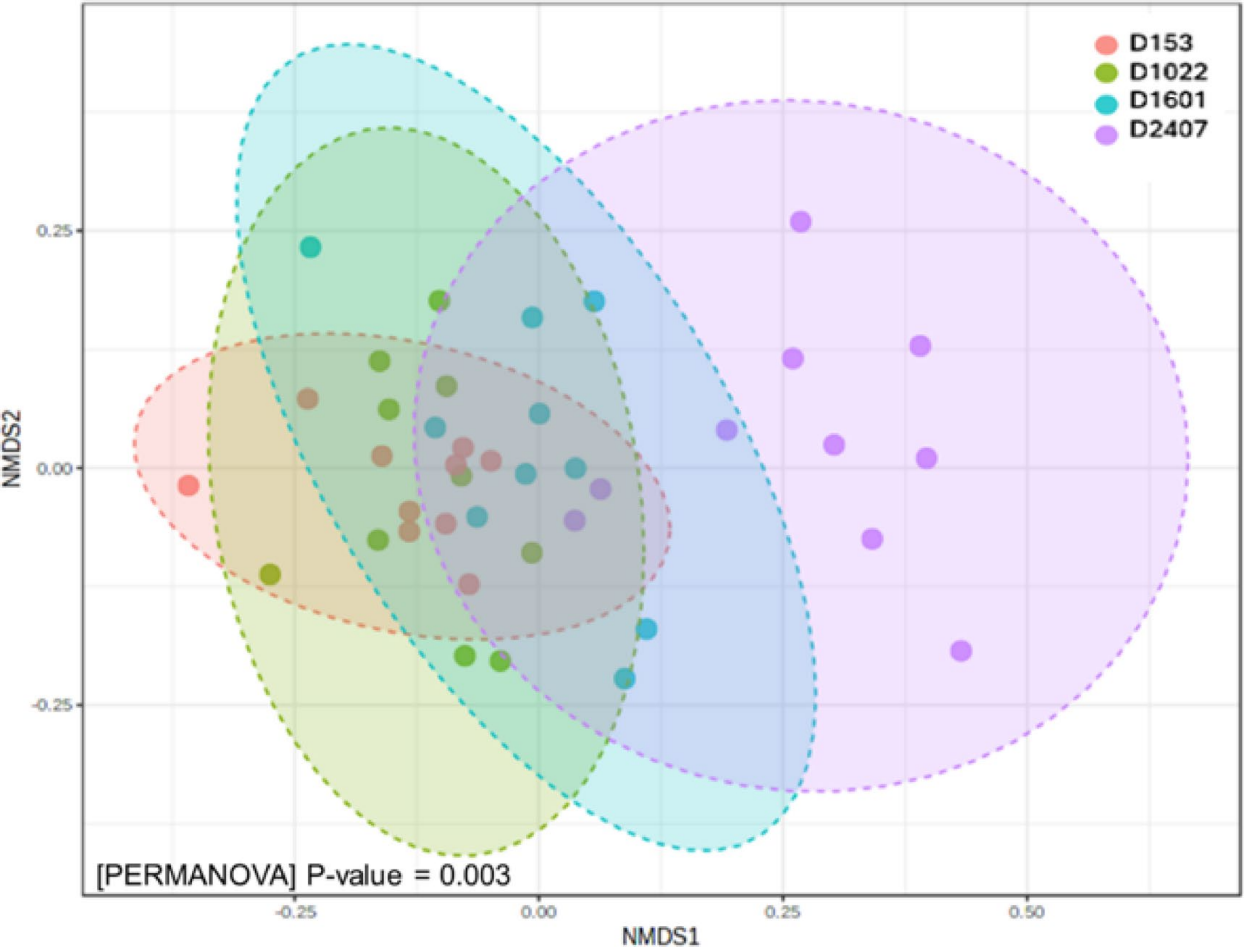
Beta-diversity analysis of the faecal microbial profile shown as a Nonmetric Multidimensional Scaling (NMDS) plot on Bray-Curtis Dissimilarity distances and differences between dietary groups using PERMANOVA.

No distinct grouping was observed for microbial communities associated with D153, D1022, D1601 and D2407, respectively. However, as presented in Fig. 3, several samples from pigs receiving 2407 ppm ZnO were visually separating in the direction of NMDS1, indicating a difference in microbial communities in the animals receiving high levels of ZnO (2407 ppm). Permutational MANOVA analysis of the distance matrix revealed a significant dissimilarity between the groups (*p* < 0.001) R^2^ = 0.204 (Fig. 3). Pairwise analysis revealed significant differences only between 2407 ppm ZnO and the other three ZnO levels (Table 1).

**Table 1:** PERMANOVA pairwise analysis of diets containing 153, 1022, 1601 and 2407 ppm ZnO (D153, D1022, D1601 and D2407, respectively) with multi-testing adjustment based on the Benjamini-Hochberg procedure (FDR).

Pair	F-value	*R*-squared	*P*-value	FDR
D153 vs. D1022	0.998234	0.0525435	0.450	0.540
D153 vs. D1601	1.71981	0.0872121	0.056	0.084
D153 vs. D2407	4.88258	0.213375	0.001	0.006
D1022 vs. D1601	0.750938	0.040048	0.694	0.694
D1022 vs. D2407	2.69863	0.130377	0.002	0.006
D1601 vs. D2407	2.35536	0.115712	0.011	0.022

**Fig. 4:**
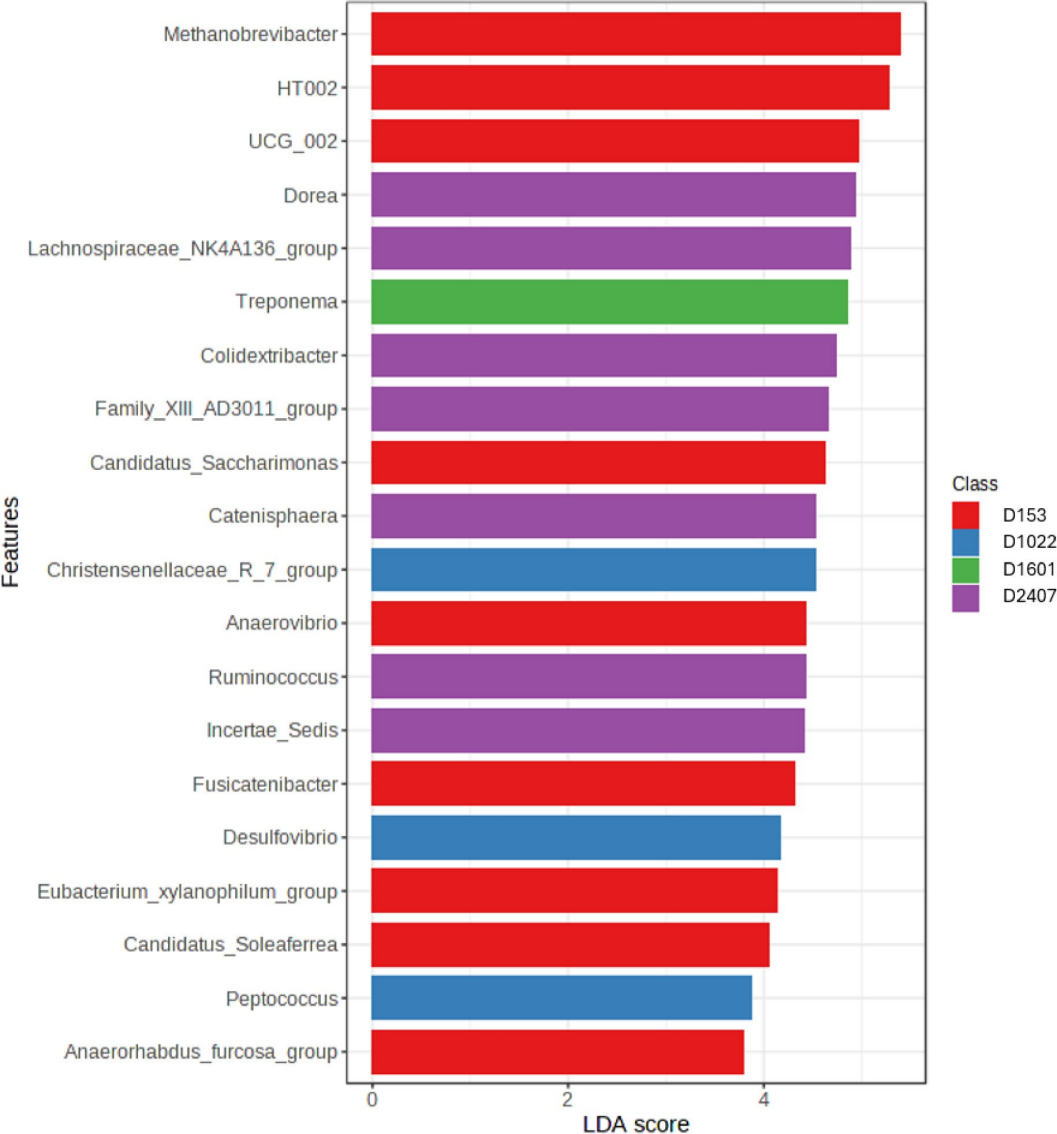
Linear Discriminant Analysis Effect Size (LEfSe) of faecal microbial features at the genus level. Bar colours correspond to the dietary groups that had the highest abundance of the respective microbial feature.

Comparisons between bacterial taxa were performed by Linear discriminant analysis Effect Size analysis (LEfSe) as seen in Fig. 4. The analysis incorporates statistical significance with biological consistency (effect size) and identifies features with significant differential abundance regarding the dietary Zn levels and the effect size of each differentially abundant feature (LDA). Features were considered significant based on the FDR adjusted *P* value (*p* < 0.05) and LDA score > 1.5. With increasing level of dietary Zn, a gradual and significant decrease in microbial abundance was observed in species from the genus: *Methanobrevibacter*, *HT002*, *Candidatus-Saccharimonas*, *Fusicatenibacter*. On the other hand, some microbial features only showed a significant decrease in abundance with 2407 ppm ZnO, such as *HT002*, *Eubacterium xylanophilum group*, *Anaerorhabdus fucosa group*, *Anaerovibrio*, *Canidatus Soleaferrea*, *Christensenellaceae R7 group*, *Desulfovibrio*, *Treponema*, *Peptococcus*. As an opposite effect, 2407 ppm ZnO led to a higher relative abundance of species from the groups: *Dorea*, *Lachnospiraceae NK4A136 group*, *Colidextribacter*, *Family XII AD3011 group*, *Catenisphera*, *Ruminococcus*, *Incertae Sedis*.

**Fig. 5:**
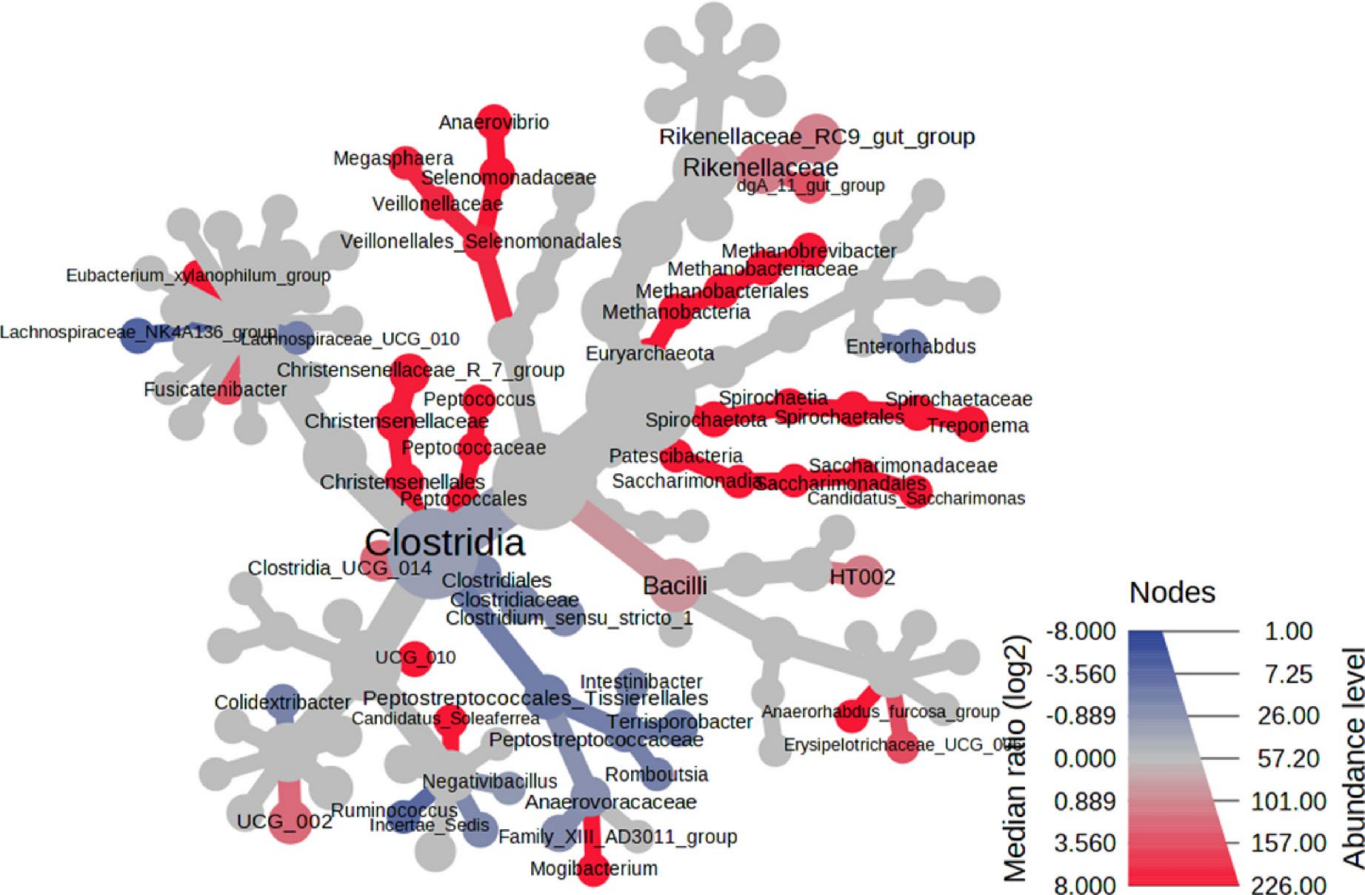
Heat tree analysis of the faecal microbial communities demonstrating the most significant differences between the lowest and highest level of dietary Zn (D153 vs. D2407).

The most significant differences in the faecal microbial communities between the lowest and highest level of Zn (D153 vs. D2407) are depicted in the heat tree analysis shown in Fig. 5. The analysis uses the hierarchical structure of taxonomic classifications (Family to Genus) to quantitatively (median abundance) and statistically (Wilcoxon Rank Sum test) visualize differences in the faecal microbial communities based on dietary Zn level. Microbial features highlighted in red (and their respective taxonomic classifications) correspond to features that have decreased significantly as a result of the high Zn level in D2407, including, members of the *Methaobacteria* community (*Methabobacteriales*, *Metahnobacteriaceae*, *Methanobrevibacter*), *Patescibacteria* community (*Patescibacteria*, *Saccharimonadia*, *Saccharimonadales*, *Saccharimonadaceae*, *Candidatus Saccharimonas*) or *Spirochaetota* community (*Spirochaetia*, *Spirochaetales*, *Spirochaetaceae*, *Treponema*). Microbial features that were increased in abundance due to the high levels of Zn are highlighted in blue, such as members of the *Clostridia* community (*Clostridiales*, *Clostridiaceae*, *Clostridium sensu stricto 1*).

### Effect of dietary Zn level on the plasma metabolite profile

**Fig. 6:**
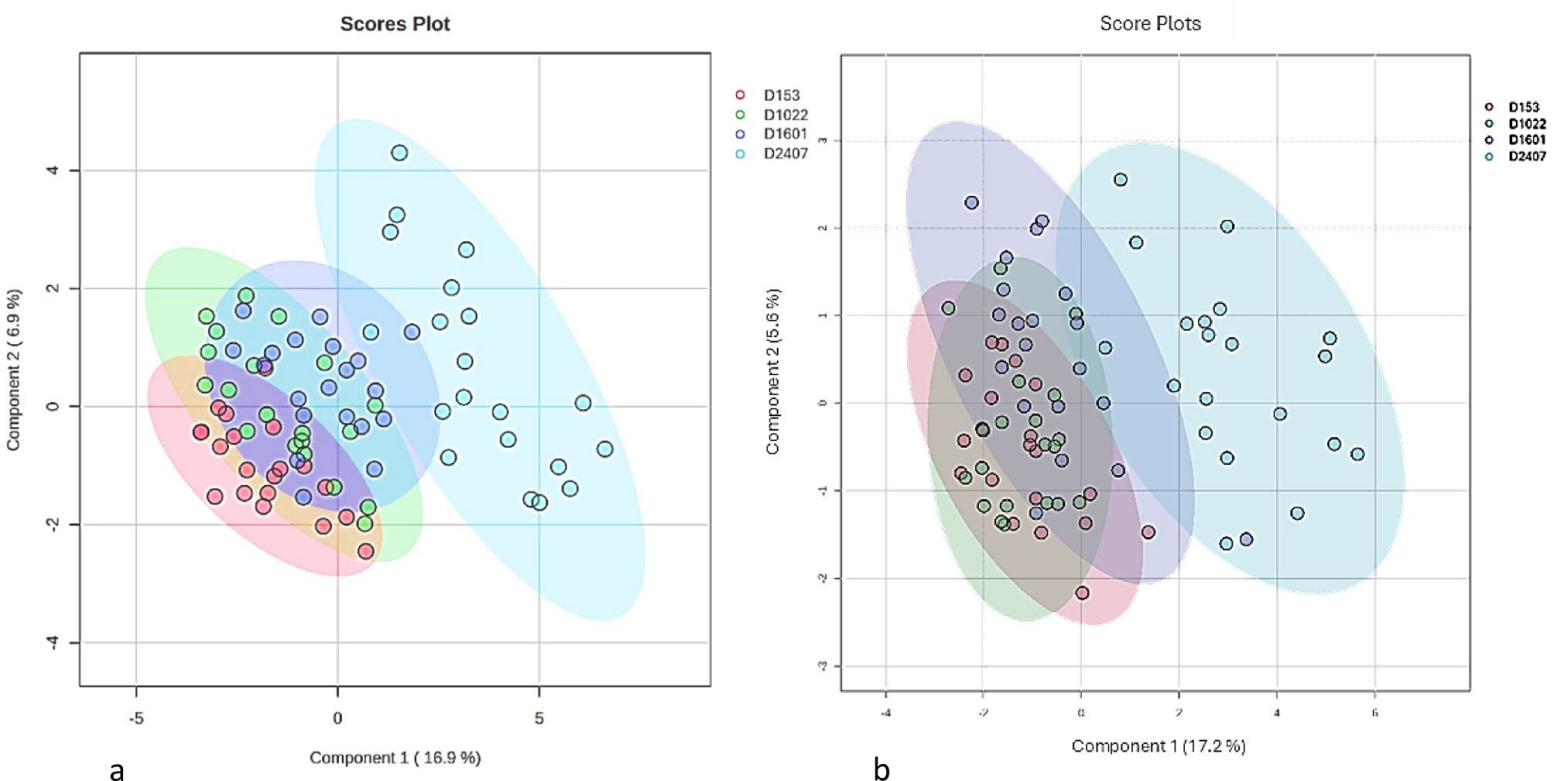
PLS-DA score plots for both positive (**a**) and negative (**b**) modes for the metabolomics profiles of plasma from pigs fed increasing dietary Zn levels.

Evaluating principal component analyses (PCA) score plots showed no visible separation between D153, D1022 and D1601 but a visible separation of D2407 to the lower dietary Zn levels (Fig. 2S). The PC1 and PC2 explained 17.6% and 13.2% of the variation in the samples, respectively. Partial Least Squares Discriminant Analysis (PLS-DA) showed a distinct grouping between D2407 compared to the rest of the diets where no grouping was visible for D153, D1022, and D1601, as presented in Fig. 6.

The key compounds responsible for differentiation between pigs receiving D153 and D2407, were identified using the multivariate analysis method of PLS-DA and the criteria including VIP > 1, *P* < 0.05, and one-way ANOVA *P* < 0.05. As presented in Table 2, a total of 26 metabolites and their fragments were identified, of which 23 were reduced by D2407 compared to D153, and 3 of them were enriched in pigs fed D2407 compared to D153. The metabolites included amino acids and intermediate metabolites of amino acids’ metabolism, microbial metabolites of branched-chain amino acids, glucuronidated compounds, phosphatidylcholines and lysophosphatidylcholines. The D2407 was associated with a significant decrease in 6-hydroxyindoxyl sulfate (8-fold), 5-hydroxy-6-methoxyindole glucuronide (5.6-fold), Indole-3-carboxaldehyde (4.9-fold), 2-phenylethanol glucuronide (2.5-fold), phosphatidylcholines (2.3-fold), lysophosphatidylcholines (2-fold), 4-ethylphenylsulfate (2-fold), hippuric acid (1.8-fold), hydroxyphenyl-acetylglycine (1.8-fold), phenylalanyl-hydroxyproline (1.7-fold), glutamylphenylalanine and D-phenylalanine (1.4- & 1.3-fold) and in a slight increase in keto-leucine (0.8-fold), 3-indoxyl sulfate (0.7-fold), and pyrocatechol sulfate (0.5-fold).

**Table 2:** Identification results of compounds in plasma discriminating between pigs fed the D2407 relative to the D153.

IM^1^	M/Z^2^	RT^3^	Adduct	Metabolite	KEGG^4^	HMDB^5^	Pathway	ID level^6^	Fold Change^7^	ANOVA (*p*-Value)
NEG	230.0129	3.90	[M-H]-	N-(2-Hydroxyphenyl) acetamide sulfate (HPAA sulfate)		HMDB0240381	Microbial metabolites of benzoxazinoid	2	8.8	< 0.001
NEG	226.018	4.78	[M-H]-	6-Hydroxyindoxyl sulfate		HMDB0240651	Microbial metabolite of tryptophan	2	8.0	< 0.001
NEG	338.0883	3.23	[M-H]-	5-Hydroxy-6-methoxyindole glucuronide	C03033	HMDB0010363	Waste/toxic compounds excretion	2	5.6	< 0.001
NEG	297.098	4.88	[M-H]-	2-Phenylethanol glucuronide	C03033	HMDB0010350	Waste/toxic compounds excretion	2	2.5	< 0.001
NEG	554.3467	10.40	[M + CH3COO]-	PC (0:0\/16:0)		HMDB0240262	Lipid metabolism	3	2.3	< 0.001
NEG	201.0229	5.18	[M-H]-	4-Ethylphenylsulfate		HMDB0062551	Microbial metabolite of tyrosine	2	2.0	< 0.001
NEG	178.0511	3.64	[M-H]-	Hippuric acid	C01586	HMDB0000714	Phenylalanine metabolism	1	1.8	< 0.001
NEG	208.0617	2.70	[M-H]-	Hydroxy phenyl acetyl glycine	C05596	HMDB0000735	Phenylalanine metabolism	2	1.8	< 0.001
NEG	277.1196	2.56	[M-H]-	Phenylalanyl-Hydroxyproline		HMDB11176	Phenylalanine metabolism	2	1.7	< 0.001
NEG	588.3308	9.36	[M + CH3COO]-	LysoPC (0:0/20:4)		HMDB0010395	Lipid metabolism	3	1.6	< 0.001
NEG	187.0976	5.00	[M-H]-	Azelaic acid	C08261	HMDB0000784	Lipid metabolism	2	1.6	< 0.001
NEG	498.2895	6.04	[M-H]-	Tauroursodeoxycholic acid		HMDB0000874	Bile acid	2	1.45	0.06
NEG	144.1032	2.64	[M-H]-	2-Aminoheptanoate		HMDB0094649	Amino acid metabolism	2	1.5	0.003
NEG	189.0402	1.21	[M-H]-	2-O-Methylascorbic acid		HMDB0240294	Ascorbic acid metabolism	2	1.2	0.003
NEG	129.0558	3.70	[M-H]-	Keto-leucine	C00233	HMDB0000695	Valine, leucine, and isoleucine degradation	2	0.8	0.004
NEG	360.0857	3.18	[M-H]-	Unidentified				4	0.7	< 0.001
NEG	212.0023	3.70	[M-H]-	3-indoxyl sulfate		HMDB0000682	Tryptophane metabolism	2	0.63	< 0.001
NEG	188.9864	3.06	[M-H]-	Pyrocatechol sulfate		HMDB0059724		2	0.5	< 0.001
POS	146.0602	3.82	[M + H]+	Indole-3-carboxaldehyde	C06324	HMDB0243900	Microbial metabolite of tryptophan	2	4.9	< 0.001
POS	180.0657	3.64	[M + H]+	Hippuric acid	C01586	HMDB0000714	Phenylalanine metabolism	1	1.8	< 0.001
POS	245.1499	2.01	[M + H]+	Hydroxyprolyl-Leucine		HMDB0028867	Amino acid metabolism	2	1.9	< 0.001
POS	279.1343	2.59	[M + H]+	Phenylalanyl-Hydroxyproline		HMDB0011176		2	1.7	< 0.001
POS	146.1178	2.68	[M + H]+	2-Aminoheptanoate		HMDB0094649		2	1.5	< 0.001
POS	144.1021	2.61	[M + H]+	Unidentified				4	1.9	< 0.001
POS	295.1292	3.26	[M + H]+	Glutamylphenylalanine		HMDB0000594		2	1.4	< 0.001
POS	166.0865	2.24	[M + H]+	D-Phenylalanine	C02057	HMDB0250791	Amino acid metabolism	2	1.3	< 0.001
POS	441.1873	2.61	[M + H]+	Unidentified				4	1.9	< 0.001
POS	510.3561	10.40	[M + H]+	LysoPE(20:0/0:0)		HMDB0010390		3	2.0	< 0.001
POS	542.3246	8.79	[M + H]+	LysoPC (20:5/0:0)		HMDB0010397		2	1.7	< 0.001
POS	544.3404	9.36	[M + H]+	LysoPC (20:4/0:0)		HMDB0010395		3	1.4	< 0.001
POS	566.3224	9.36	[M + Na]+	LysoPC (20:4/0:0)		HMDB0010395		3	1.4	< 0.001

^1^IM: Ionisation mode.

^2^M/Z: Mass to charge ratio.

^3^RT: Retention time.

^4^KEGG: Kyoto Encyclopedia of Genes and Genomes^56–58^.

^5^HMDB: Human Metabolome Database (https://hmdb.ca/).

^6^ID levels: Level 1: Identified using authentic standards; Level 2: Putatively annotated compounds Annotation verified using MS/MS libraries; Level 3: Putatively characterised compound classes; Level 4: Unknown or unidentified.

^7^Fold change: is calculated as the log transformation of the ratio between the mean metabolite abundance in the D2407 relative to the D153. Values above 1 show reduction of the metabolite in D2407 and values below 1 show enriched metabolites in D2407.

### Correlations between faecal microbiota and discriminating plasma metabolites

**Fig. 7:**
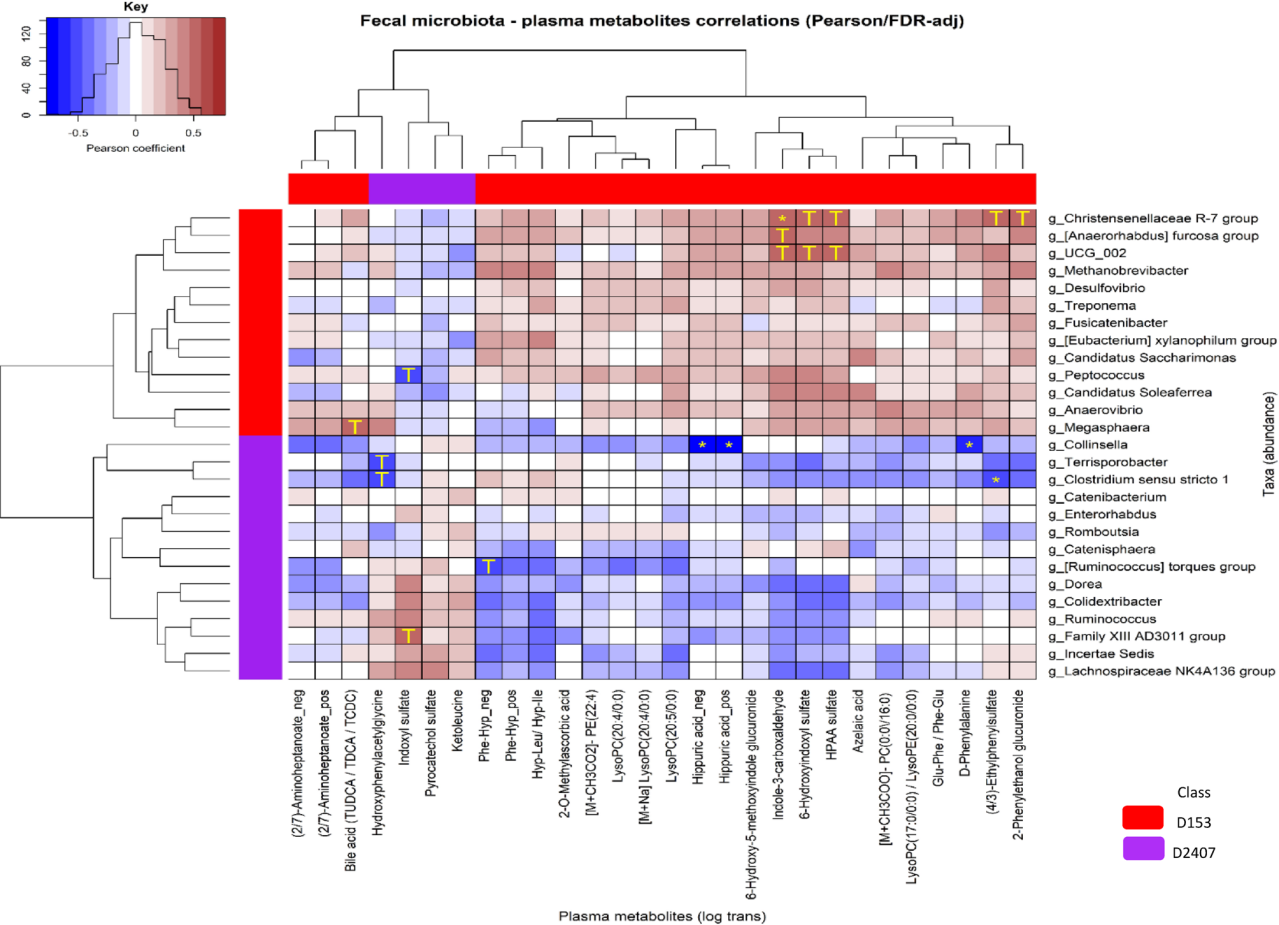
Correlations between the dominant faecal bacteria at the genus level and plasma metabolite intensities. Statistically significant correlations are presented, coloured according to the Pearson coefficient distribution: red represents a positive correlation (*p* < 0.05), blue represents a negative correlation (*p* < 0.05), and white indicates a non-significant correlation (*p* > 0.05). Stars represent significant correlation (*p* < 0.05), and T represents tendencies (0.05 < *p* < 0.1).

The heat map illustrated in Fig. 7 shows a Pearson’s correlation analysis between metabolite intensities and the LEfSe selected bacterial taxa responsible for differentiation between D153 and D2407. The x-axis contains plasma metabolites that were significantly altered between D153 and D2407 according to PLSR analysis. Yellow stars indicate statistically significant differences according to the Pearson’s test with false discovery rate correction, whereas yellow T indicate tendencies (*p* = 0.05–0.1).

We observed that at genus level, the abundance of *Christensenellaceae R7 group* were positively correlated withindole-3-carboxaldehyde (*p* < 0.001) and had a positive correlation tendency with 2-phenylethanol glucuronide (*p* = 0.073), 4-ethylphenylsulfate (*p* = 0.073), HPAA sulfate (*p* = 0.059), and 6-hydroxyindoxyl sulfate (*p* = 0.065). The abundance of *Collinsella* were negatively correlated with D-phenylalanine and hippuric acid (*p* < 0.001). The abundance of *Clostridium sensu stricto 1* were negatively correlated with 4-ethylphenylsulfate (*p* < 0.001) and had a tendency for negative correlation with hydroxy phenyl acetyl glycine (*p* = 0.076). The abundance of *Anaerorhabdus fucosa group* and *UCG_002* had positive correlation tendencies with indole-3-carboxaldehyde. Additionally, 6-hydroxyindoxyl sulfate and 2-acetamidophenyl sulfate (other names include: N-(2-hydroxyphenyl) acetamide sulfate and HPAA sulfate) also showed a positive correlation tendency with *UGC_002* (*p* = 0.073). *Peptococcus* abundance had a negative correlation tendency with indoxyl sulfate (*p* = 0.065) and bile acids including taurochenodeoxycholic acid (TCDC), tauroursodeoxycholic acid (TUDCA), and taurodeoxycholic Acid (TDCA) showed a positive correlation tendency with *Megasphaera* (*p* = 0.065). Hydroxy phenylacetyl glycine had a negative correlation tendency with the abundance of *Terrisporobacter* (*p* = 0.065) and *Clostridium sensu stricto 1* (*p* = 0.076). Indoxyl sulfate had a positive correlation tendency with the abundance of *Family XIII AD3011 group* (*p* = 0.092). Finally, a tendency to a negative correlation between *Ruminococcus torques group* and phenylalanyl-hydroxyproline (*p* = 0.077) was observed.

## Discussion

Previously, we reported that approximately 1400 ppm dietary Zn was required for optimal feed intake and growth during the first two weeks after weaning^6^, which is much higher than NRC´s current recommended dietary Zn content (80–100 ppm) for pigs between 6 and 25 kg of body weight^5^. Earlier, Zn supplementation from ZnO in doses at or above 2500 ppm has been designated “pharmacological” meaning that it induced positive effects in the animal beyond covering the actual Zn requirements as a micronutrient^16^. However, there seems to be no simple way to distinguish between the dietary Zn level as a micronutrient and the level of Zn as a “pharmaceutical” agent.

There is a plethora of studies investigating the effects of pharmaceutical Zn levels in relation to PWD, mostly reporting improved growth performance, nutrient digestibility, immune response, intestinal morphology and integrity, and hindgut microbiota composition. However, the changes in microbial functional properties, links to the host animal’s metabolism, and molecular modes of action of pharmaceutical ZnO as a dietary strategy to manage PWD remain mostly unknown^8,10,17^. Prior research has found that pharmaceutical ZnO levels does not enhance growth performance in germ-free animals and the positive impacts of high ZnO supplementation may originate exclusively from modulatory effects of Zn on gut microbiota^18,19^. In the present study we corroborated this aspect and demonstrated that only the highest dietary ZnO level (2407 ppm) resulted in changes of faecal microbial population and composition and plasma metabolic profile of pigs three weeks after weaning, indicating that a pharmaceutical effect of dietary Zn only is apparent in the highest Zn level (2407 ppm).The other two intermediate Zn levels showed little, or no differences compared to low Zn diet (153 ppm).

In line with previous reports, our data showed that D2407 reduced faecal microbial richness in pigs, indicating the strong effects of high dietary Zn on the number of different bacteria comprising faecal microbiota^12^. However, in our study, no effects of high dietary Zn were detected on bacterial diversity addressed by the Simpson’s index. This is similar to some^12,19^ but not all previous studies, where pharmaceutical levels of ZnO resulted in reduced microbial diversity^20^, perhaps due to differences in the employed methodologies, DNA extractions or bioinformatic tools used in the study of the microbial flora.

In the current study, both *Firmicutes* and *Bacteroidetes* were the two dominant phyla at all dietary Zn levels, followed by *Actinobacteriota*, which was in line with previous reports^14,19,21^. *Firmicutes* and *Bacteroidetes* are the two dominant phyla of bacteria commonly found in the gut microbiota of pigs and other mammals contributing to the overall health of the animal^22^. *Firmicutes* has been reported to be involved in energy haemostasis in the body and feed efficiency of pigs^23^. Numerous members within this phylum and *Bacteroides* are reported to produce butyrate and other short-chain fatty acids, which enhance immune responses by activating T cells and therefore restricts gastrointestinal (GI) tract colonisation by harmful bacteria^24^.

The D2407 reduced the abundance of bacteria belonging to *Euryarchaeota*, *Spirochaeota*, *Desulfobacterota*, and *Patescibacteria* taxa. These include members of the *Methanobacteria* community (*Methanobacteriales*, *Methanobacteriaceae*, *Methanobrevibacter*), *Patescibacteria* community (*Patescibacteria*, *Saccharimonadia*, *Saccharimonadales*, *Saccharimonadaceae*, *Candidatus Saccharimonas*) or *Spirochaetota* community (*Spirochaetia*, *Spirochaetales*, *Spirochaetaceae*, *Treponema*).

The *Euryarchaeota*, a phylum of archaea, includes many *Methanogens* primarily found in pigs during later stages of growth, and exhibits variations in relative abundance that are largely influenced by dietary composition^25^. *Methanogens* can produce methane through cell wall polysaccharide (cellulose) degradation and has been reported to be associated with high feed efficiency in pigs^26^. The *Spirochaeota* is a phylum containing many pathogenic bacteria such as *Treponema*, which was significantly reduced by increasing dietary Zn levels and disappeared at 2407 ppm ZnO. A previous study has reported the links between *Spirochaetes* phylum and damaged enterocytes and mucus production in the intestinal lumen and caecum of sows with swine dysentery^27^.

*Desulfovibrio* being the most prevalent species of sulfate-reducing bacteria produces large quantities of hydrogen sulphide from sulfur-containing amino acids such as methionine and cystine^28^, and has been reported to be associated with gut inflammation^29^. Hydrogen sulfide has opposing effects at low vs. high concentrations. At high concentrations, it induces intestinal inflammation and is associated with PWD^30^. In our study, members of *Desulfobacterota* phylum such as *Desulfovibrio* was reduced by the highest dietary Zn level (2407 ppm). At genus level, members of the *Clostridia* community including *Clostridiales*, *Clostridiaceae*, and *Clostridium sensu stricto 1* were increased in abundance at the highest dietary Zn level. The majority of *Clostridium* species are saprophytic, meaning they break down plant polysaccharides and produce short-chain fatty acids^31^. The increased abundance of butyrate-producing bacteria in the hind-gut of piglets fed 2407 ppm ZnO, may help reduce pH and exclude other pathogenic bacteria^14^, such as *Desulfovibrio*, which were reduced at 2407 ppm ZnO.

The abrupt change in diet at weaning can lead to shifts in the relative abundance of different microbial taxa in the gut. Certain bacteria, such as *Lactobacillus* and *Bifidobacterium*, which are commonly found in milk-fed piglets, may decrease in abundance, while others, such as *Prevotella* and *Fibrobacter*, associated with fiber digestion, may increase as piglets adapt to a solid plant-based diet^32,33^. Disruption of the beneficial bacteria and overgrowth of harmful pathogens such as *Escherichia coli*, *Clostridium perfringens*, or *Brachyspira species* can lead to PWD. Our results provide clear evidence that only the highest dietary Zn level reduces the abundance of pathogenic bacteria in the hindgut of piglets three weeks after weaning and encourages the increased abundance of beneficial bacteria.

Few studies have investigated integrated gut microbiota and molecular functions such as transcriptomics in pigs when supplemented with high dietary Zn to prevent PWD. In a recent study by Schokker et al. (2023), the effects of both low (100 ppm) and high (2690 ppm) dietary Zn levels, on gut tissue gene expression and gut microbial profile were investigated in clinically healthy pigs after weaning. The authors reported changes in small intestinal functionality, inter-change in commensal bacteria, and upregulation of gut tissue genes expression, mostly involved in immunity and inflammatory responses, in the piglets receiving high dietary Zn. It was hypothesized that the effects of commensal bacteria on gut tissue gene expression relevant to immune and inflammatory responses could be induced by specific microbial metabolites^59^. Gut microbiota and gut-derived metabolites have been reported to influence neurotransmitter production and impact metabolism as the intricate molecular communication between mammals and the vast number of microbial cells inhabiting their digestive systems is fundamental to their symbiotic relationship^34^. Zhang et al., (2019) fed piglet with low (20 mg/kg) and high (300 mg/kg) Cu sulfate levels and studied the hindgut microbiome-metabolome responses in Suhuai suckling piglets and reported clear shifts in the relative abundance of several genera such as *Clostridia* and butyrate-producing bacteria. These microbial shifts were associated with changes in carbohydrate and protein metabolism. They reported positive or negative correlations among specific bacterial families with energy or protein metabolism, providing a foundation on the effects of nutrition in altering the composition of gut microbiota and modulating microbial metabolites in the intestine^14^.

The metabolomics data revealed that pigs receiving 2407 ppm ZnO had a distinct blood metabolic profile compared to pigs receiving the other 3 levels of dietary Zn. Among the identified metabolites responsible for the grouping to the Zn levels were the 6-hydroxyindoxyl sulfate, which was decreased considerably in pigs fed 2407 ppm ZnO. 6-hydroxyindoxyl sulfate is a metabolite of tryptophan breakdown, and formed in the body exclusively by gut microbiota as it is not detected in microbiota-free gnotobiotic mice^35^. Products of tryptophan metabolism, indole compounds produced by gut bacteria via tryptophanase^34^, may undergo further hepatic transformation, which can lead to the formation of 6-hydroxyindoxyl sulfate. 6-hydroxyindoxyl sulfate (or indole sulfate), which has been studied for its potential role in various physiological and pathological processes, is a uremic toxin and is likely to be linked to gut inflammation and PWD. More than 85 gram-positive and gram-negative species such as *Lactobacillus*,* Bifidobacterium longum*,* Bacteroides fragilis*,* Parabacteroides distasonis*,* Clostridium bartlettii*,* E. hallii* are known to hydrolyse tryptophan to indole via tryptophanase, making them the most prevalent microbial tryptophan catabolites^34,36^.

Not every indole compound is harmful. While some indole derivatives produced by pathogenic bacteria may act as virulence factors, contributing to the development of infections or inflammatory diseases, other indole compounds, such as indole acetic acid, indole acrylic acid, indole-3-carboxaldehyde, tryptamine, indole propionic acid, and indole lactic acid produced by gut bacteria such as *Clostridium sporogenes* and *Lactobacillus*, are reported to enhance gut motility^37^, modulate immune responses and induce anti-inflammatory and antioxidant properties^38^, and facilitate mucosal homeostasis, maintaining intestinal barrier integrity and function^39^.

The dual nature of microbial indole compounds underscores the complexity of host-microbe interactions in the GI tract. While some compounds may pose risks to host health under certain conditions, others may contribute to host well-being and play essential roles in maintaining host-microbe symbiosis^40^.

4-ethylphenyl sulfate is a microbial metabolite of tyrosine, a precursor of several mammalian neurotransmitters. Gut bacteria convert tyrosine to 4-ethylphenol, which is then sulphated to 4-ethylphenyl sulfate by the host. Studies in germ-free mice have reported no detectable 4-ethylphenyl sulfate levels in the body^41^.

In the present study, Pearson correlation analysis showed positive correlation between the *Christensenellaceae R-7 group* and indole-3-carboxaldehyde, 2-phenylethanol glucuronide, 4-ethylphenylsulphate, HPAA sulfate, and 6-hydroxyindoxyl sulfate. The abundance of *Christensenellaceae R7 group* and the relative intensity of the correlated metabolites were decreased in pigs fed D2407. Additionally, 4-ethylphenyl sulfate was negatively correlated with the abundance of *Clostridium sensu stricto 1*, a member of *Clostridia* community, which was increased in abundance in pigs fed the D2407. The abundance of *Clostridium sensu stricto 1* was also negatively correlated with hydroxy phenylacetyl glycine, which has been identified as a gut microbial metabolite of phenylalanine. Although no relevance to gut inflammation has been identified yet, however, it has been reported that phenylacetic acid can effectively cause liver steatosis and initiate the metabolism of branched-chain amino acids^42^.

Our Pearson correlation analysis revealed a significant negative correlation between the abundance of *Collinsella* with D-phenylalanine and hippuric acid. *Collinsella*, a genus of *Actinomycetota*, known for its role in metabolizing certain dietary compounds, particularly bile acids and carbohydrates producing short-chain fatty acids, have been reported to increase in abundance following low fibre diets by using non-fibre energy sources, such as host mucus glycoproteins. This may lead to erosion of the colonic mucus barrier^43^. Hippuric acid is another bacterial metabolite of phenylalanine metabolism reported to have a positive correlation with microbial diversity^44^.

Similar to Zn, excessive Cu is often also supplemented in piglet diets to help manage PWD. The microbiome-metabolome responses to high dietary Cu levels in the hindgut of suckling piglets have revealed a clear decrease in the abundance of several *Clostridia* genera and relative abundance of butyrate-producing bacteria, such as *Coprococcus*, *Roseburia*, and *Acidaminococcus*. These microbial shifts were associated with changes in carbohydrate and sugars’ metabolism including galactose metabolism and gluconeogenesis, urea cycle, protein biosynthesis, and amino acids’ metabolism including arginine, proline, β-alanine, phenylalanine, tyrosine, and methionine. Among the microbial metabolites of the aromatic amino acids, several indoles, hippuric acid, hydroxy phenylacetyl glycine, 4-ethylphenylsulphate, HPAA sulfate, phenyl sulfate were correlated with specific bacterial species such as *Christensenellaceae R7 group*, *Clostridium sensu stricto 1*, *Collinsella*, which were altered by the D2407 in the current study. These findings are well aligned with previous studies indicating an association between gut health and immune status of piglets and the composition of the gut microbiota and their respective blood metabolites. The incidence of PWD was correlated with the decarboxylation of amino acids and production of amines by specific intestinal microbiota such as *Acidaminococcus*^14^.

## Conclusions

The mechanism of action for pharmaceutical levels of ZnO (approximately 2400 ppm) in the diet appears to involve reducing the production of toxic and inflammatory microbial compounds, such as 6-hydroxyindoxyl sulfate and 4-ethylphenyl sulfate, by decreasing the abundance of pathogenic bacteria like *Christensenellaceae R7 group*, *Desulfovibrio*, *Treponema*, and *Peptococcus*. At the same time, pharmaceutical ZnO levels may improve the digestion and utilization of cell-wall carbohydrates in weaning pigs by promoting beneficial bacteria involved in fiber fermentation and short-chain fatty acid production, including *Lachnospiraceae*, *Ruminococcus*, and *Clostridia*.

Key microbial metabolites, such as indole compounds, play dual roles depending on bacterial types and end-products, acting as signalling molecules in the gut-brain axis. Further research is needed to better understand the factors driving the production of toxic versus beneficial indole compounds and to target specific microbial pathways for improved gut health. Leveraging microbiota-derived metabolites offers a promising strategy for enhancing gut health and host physiology.

## Methods

### Ethics approval

All experimental procedures involving the use of animals complied with the “Danish Ministry of Justice, Law no. 474/15.05.2014 concerning animal experiments and care and license issued by the Danish Animal Experiments Inspectorate, Ministry of Food, Agriculture and Fisheries, the Danish Veterinary and Food Administration. All animal experiments were carried out in full compliance with the ARRIVE guidelines and reported in accordance with the applicable regulations and standards set by the National Institutes of Health.

### Animals, housing, diets, and sampling

The present study used a subset of samples obtained from a larger Zn dose-response experiment investigating the Zn requirement in piglets during the first three weeks after weaning^6^. In the original study, a total of 180 crossbred ([Danish Landrace × Yorkshire] × Duroc) pigs (half female, half castrates) were sourced from a commercial piggery and transferred to the research facility on the day of weaning (28 days of age). Pigs (7.63 ± 0.98 kg of body weight) were randomly assigned according to body weight and sex to six dietary Zn levels (*n* = 30 per level) from weaning day 0 to day 21 and individually housed (details can be found in^6^. After the completion of the trial, pigs were transferred to the fattening unit and fed until 110 kg to be sent to abattoir.

The experimental diets were produced by adding Zn in the form of high purity (80%) ZnO (VetZink, Vepidan Aps, Løgstør, Denmark) to a basal diet to supply 100, 450, 950, 1450, 1950, and 2450 ppm corresponding to 155 (D155), 493(D493), 1022 (D1022), 1601 (D1601), 2052 (D2052), and 2407 (D2407) ppm analysed total Zn in the diets (Table 3). The basal diet met the Danish recommendations for all nutrients except for Zn, which was excluded from the mineral-vitamin premix. The response criteria included weight gain, feed intake, daily visual diarrhea score and serum Zn concentration.

**Table 3:** Ingredients and calculated crude protein, lysine and mineral composition of the basal diet.

Ingredients	Basal Diet	D153	D1022	D1601	D2407	
Wheat	40.3					
Barley	20.0					
Soy protein concentrate, HP300	9.8					
Soybean meal, 45.8% protein	8.0					
Oats	5.9					
Vegetable fat and oil	4.8					
Lactose	4.1					
Fishmeal	3.0					
Vitamin/mineral premix^2^	2.2					
Monocalcium phosphate	1.1					
Salt	0.7					
Aroma	0.1					
Natuphos 10,000 E^1^	0.02					
**Calculated composition (as-fed)**						
Crude protein, %	18.3					
Lysine, %	1.25					
Ca, %	0.74					
P, % (available)	0.40					
Fe, mg/kg	180					
Zn, mg/kg	29					
Cu, mg/kg	120					
Added dietary zinc (ZnO), ppm	0	100	950	1450	2450	
Analysed total zinc concentration (as-fed), ppm	49	153	1022	1601	2407	

^1^200% phytase = 1000 FUT/kg, Natuphos 10,000 E.

^2^Not containing zinc.

### Faeces and blood sampling for Microbiome and non-targeted metabolomics studies

Hansen et al. (2022) concluded, that newly weaned pigs require 1400 ppm dietary Zn, corresponding to a daily intake of approximately 400 mg Zn for the first two weeks post weaning when growth, feed intake, probability of diarrhea and serum Zn status were considered^6^. For the current study, for non-targeted metabolomics analysis, we randomly chose a subset of blood samples obtained on day 21 post weaning from 80 pigs (*n* = 20 pigs/diet) receiving dietary Zn levels below what was estimated to cover the Zn requirement (D153, D1022) and from pigs receiving dietary Zn levels above what was estimated to cover the Zn requirement (D1601, D2407). Additionally, for microbial profile study, grab faeces were collected from a subset of 40 pigs (*n* = 10 pigs/ diet) also receiving D153, D1022, D1601, and D2407. The pigs selected for grab faecal sampling were randomly picked among the 80 pigs from which the blood samples were selected. Blood samples were collected from the jugular vein and plasma harvested after centrifugation (3,000 rpm for 10 min at 4^o^C) and stored at -80^o^C until analysis.

### Faecal microbial analysis

A 16 S rRNA amplicon sequencing technique was used to investigate gut microbiota of pigs. Total bacterial DNA was extracted from faecal material using a Nucleospin Stool DNA extraction kit (Machery-Nagel, Düren, Germany) as described by^45^ with the following modifications: 700 µL of lysis buffer SL2 was added to 250 mg of faces. Samples were disrupted in a FastPrep-24TM benchtop homogenizer for 2 × 20 s. DNA was eluted in a final volume of 60 µL of elution buffer. The concentration of the DNA was quantified using the qubit broad range assay kit (Thermo Fisher Scientific, Wilmington, DE, USA). Amplicon libraries covering the V3-V4 region of the 16 S rRNA gene were also prepared according to^45^ using universal primers Bac341F and Bac805R as recommended by^46^. Amplicon libraries were sequenced on the Illumina MiSeq (Illumina, San Diego, CA, USA) using 300 bp paired-end reads. First, raw sequence data were demultiplexed and quality filtered using the q2-demux plugin and the primers were removed using cutadapt in QIIME2 (qiime2 core 2019.7;^47^. Bioinformatics on sequence reads were performed in RStudio (R version 4.0.5 2021-03-31) using the DADA2 pipeline and the dada2 package (version 1.22.0) as described by ^48^ and ^49^. The sequence reads were denoised and grouped into amplicon sequence variants (ASV) with DADA2 with the following options: forward reads truncated after 280 bases and reverse reads truncated after 210 bases resulting in a feature table of amplicon sequence of variants (ASVs). Sequencing reads were dereplicated and sample inference was preformed followed by merging of paired reads for the final ASVs table. Chimeric reads were removed from the dataset using the consensus method. Taxonomy was assigned using the “assignTaxonomy” and “addSpecies” using a trained classifier based on the SILVA database (“silva_nr99_v138.1_train_set.fa.gz” and “silva_species_assignment_v138.1.fa.gz”, published March 7, 2021;^50^. Representative sequences of ASVs were used to construct a phylogenetic tree using the “phangorn” R package (version 2.11.1,^51^. All results from DADA2 were then transferred to the phyloseq package in RStudio (version 1.38.0) and used for exportation of the ASVs table, sample data information, tree, and taxonomy classifications. The exported files were used for marker data profiling in MicrobiomeAnalyst 2.0 ^52^. The dataset was further filtered to remove low abundance features (< 2 counts per sample) with a minimum prevalence in 50% of the samples. This resulted in a dataset of 226 ASVs. The data was scaled using Cumulative Sum Scaling (CSS) before calculating alpha-diversity metrics, beta diversity metrics and calculating linear discriminant analysis (LDA) effect size (LEfSe). All diversity metrics were performed on sequences at the genus level. Alpha diversity was visualized with box plots and beta diversity using Nonmetric Multidimensional Scaling (NMDS) plots and Bray-Curtis Dissimilarity Index. Group significance on alpha-diversity metrics was performed using ANOVA and on beta-diversity metrics with permutational MANOVA (PERMANOVA/adonis) with multi-testing adjustment based on Benjamini-Hochberg procedure (false discovery rate, FDR). Results were considered significant at P-value ≤ 0.05. LEfSe analysis was also performed on sequences at the genus level and thresholds were set for the most significant and abundant features of interest discriminating between the dietary groups: logLDA score of 1.5 and cutoff P-value ≤ 0.05 and P-value ≤ 0.1 for FDR-adjusted values.

A correlation analysis between selected taxonomic features and metabolites was performed in RStudio (R Version 4.1.3–2023.06.1). The genera taxonomic features were selected from the LEFSE analysis as the most significant and abundant features of interest, whereas the metabolomics data set comprised the most discriminant variables from the PLSR analysis. Intensities of metabolites were log transformed before correlation. Pearson’s test with false discovery rate P-value correction was used to determine significance by using the corr.test function and plotted using the heatmap.2 function from the R-package gplots (RStudio, R).

### Non-Targeted LC-MS metabolomics study of blood plasma

Blood plasma sample preparation for non-targeted metabolomics study was performed as previously described^53^. Briefly, metabolites were extracted from 100 µL plasma sample, deproteinized by mixing with 300 µL acetonitrile including internal standard (glycocholic acid (Glycine-1-^13^ C) and p-chlorophenylalanine, Sigma, MO) at a final concentration of 10 µg/mL. Samples were vortexed after mixing and incubated at 4 °C for 20 min. Then they were centrifuged for 10 min at 13,200 g, and the supernatant was transferred to a fresh tube and evaporated to complete dryness using a vacuum centrifuge set at 2000 rpm and 30 °C. The dry residue including extracted metabolites was resuspended in 100 µL of water/acetonitrile/formic acid (95:5:0.1, v/v/v) and centrifuged for 10 min at 1000 g. For the analysis, the supernatant was transferred to LC-MS vials containing inserts and injected to the instrument at a volume of 2 µL. An ultra performance liquid chromatography Ultimate 3000 system (UHPLC, Dionex, Sunnyvale, CA) was used to perform chromatographic separation using a C18 analytical column Acquity HSS T3 column (1.7 μm 100 × 2.1 mm, Waters Ltd., Elstree, U.K.) maintained at 30 °C. The mobile phases consisted of (A) Milli-Q H2O with 0.1% formic acid and (B) acetonitrile with 0.1% formic acid. The flow rate was 400 µL/min. Metabolites were eluted with a linear gradient starting 0.1 min after injection of the sample. The gradient rose linearly from 5 to 90% B in 11.9 min, after which the column was held in isocratic conditions for 0.3 min, and then it decreased back to 5% B in 0.2 min and the column equilibrated at 5% B for 2 min. An Impact HD quadrupole time-of-flight (QTOF) mass spectrometer (Bruker Daltonics, Bremen, Germany) was used to ionize the eluent by electrospray set in positive and negative modes to 4500 and 3600 V, respectively. The instrument setting parameters included: end plate-offset voltage at 500 V in both the positive and negative modes; the dry gas flow at 8 L/min at a temperature of 200 °C; nebulizer pressure 1.8 bar; the scan range from 50 to 1000 m/z; sampling rate of 1 Hz; Collision energy during MS was set to 6 eV; and external calibrant (lithium formate clusters) was employed in the beginning of each chromatographic run, at a concentration of 5 mM in water-isopropanol-formic acid. For MS/MS analysis, all the parameters and setting were similar to MS, however, Ar was used as the collision gas and collision energy ranged from 10 to 40 eV.

### Sample quality control and metabolomics data processing

To monitor the quality of the chromatographic runs, the stability of the system as well as the accuracy of the sample preparation, quality control (QC) samples prepared by pooling aliquots of all samples and blank samples, subjected to the same sample preparation protocol, were continuously injected throughout the batch of samples.

### LC-MS data processing

The data were processed before statistical analyses as described in detail by^54^. In brief, the calibrated mass spectra were converted into mzXML file format. Mass features of the plasma samples were extracted using the R-based XCMS package^55^. Chromatographic peak picking was done with “centWave” method and retention times were aligned using the “Orbiwarp” algorithm. “fillPeaks” function was used to substitute missing values and fragments, isotopes and adducts were automatically annotated using CAMERA. The exported data tables were subjected to cleanup to remove features present in blank (solvent contamination peaks, column, or plastic peaks from sample preparation). Finally, the retention time of the dataset was truncated to contain only the main regions of interest with chromatographic peaks.

### Multivariate data analysis

The output was exported to web-based open access MetaboAnalyst 5.0 platform for comprehensive data analysis and interpretation. Data were subjected to log10 transformation and pareto-scaling before performing principal component analysis (PCA). The score plots of PCA were inspected to identify outliers and for pattern recognition. Partial least squares (PLS) models were recalculated and loading plots of PLS model were used to detect metabolite ions with the greatest influence on the clustering of pigs. Exact mass and the mass spectrometric fragmentation features were used for identification of metabolites by comparing to the METLIN (http://metlin.scripps.edu/) and Human Metabolome Database (http://www.hmdb.ca/) online databases. When available, the putatively identified metabolites were confirmed using chemical standards following the same analytical platform and sample preparation protocols.

## Electronic supplementary material

Below is the link to the electronic supplementary material.

Supplementary Material 1

## Data Availability

The datasets generated during and/or analysed during the current study are available from the corresponding author on reasonable request.
