# Supplementary material for: Integrated faecal microbiota and blood metabolic changes following different dietary zinc oxide levels in weaned piglets
Source: Sci Rep. 2025 May 26;15:18346. doi: 10.1038/s41598-025-03103-7 (PMC12106686; doi:10.1038/s41598-025-03103-7)
Supplement: Supplementary file 1 — Supplementary Material 1 [file 41598_2025_3103_MOESM1_ESM.docx]

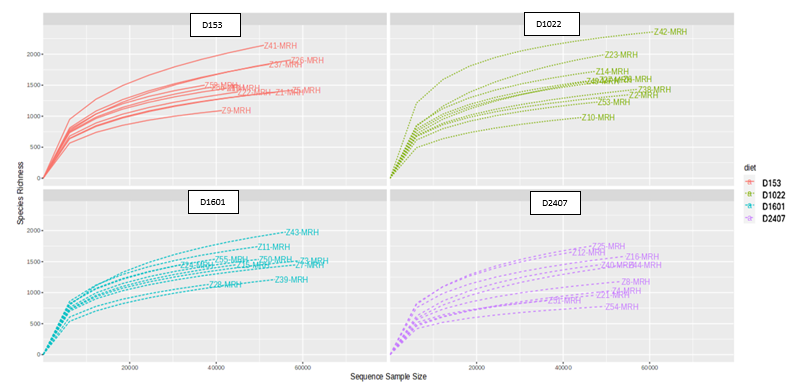


**Figure S1.** Rarefaction plot showing the total number of raw sequences reads and richness obtained per sample and dietary group.


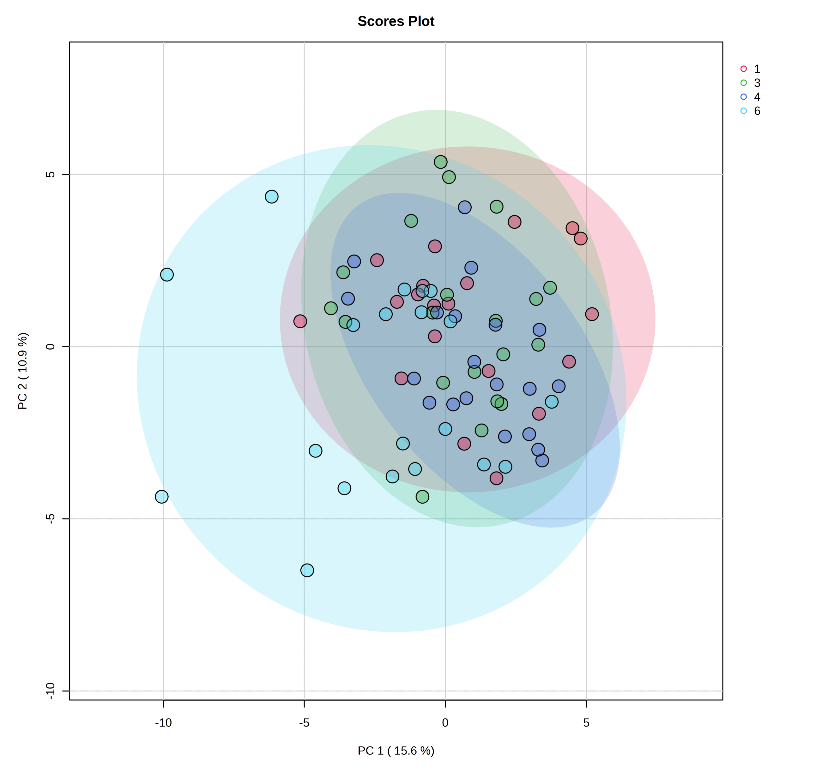


(a)

**Figure S2**. PCA Score plots, positive (a) and negative (b) modes.


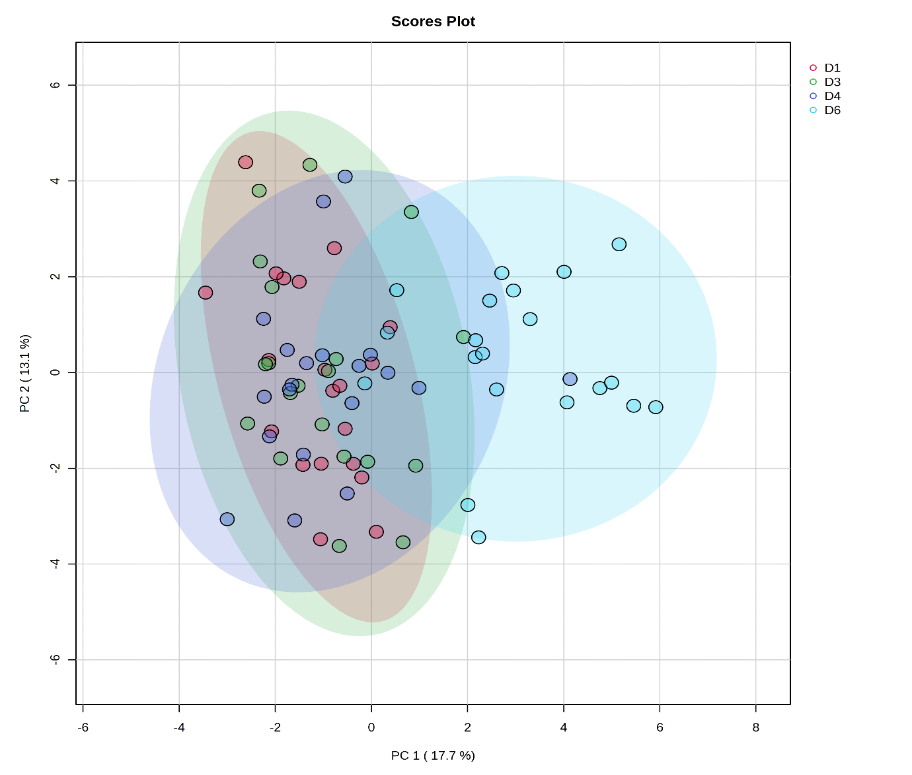


(b)

D153

D1022

D1601

D2407
